# Prediction of peri-operative mortality in care of preterm children in non-cardiac surgery

**DOI:** 10.1186/s12871-025-03168-x

**Published:** 2025-06-19

**Authors:** Gerrit Jansen, Linda Irmscher, Sunil Jagoda, Jochen Hinkelbein, Theodor W. May, Jakob Popp, Sebastian Rehberg

**Affiliations:** 1 University Department of Anesthesiology, Intensive Care Medicine, Emergency Medicine and Pain Medicine, Johannes Wesling Klinikum Minden, Ruhr University Bochum, Hans-Nolte-Straße 1, Minden, 32429 Germany; 2 Department of Anesthesiology and Intensive Care Medicine, Landeskrankenhaus Bludenz, Vlbg. Krankenhaus- Betriebsgesellschaft.m.b.h., Carinagasse 41, Feldkirch, A-6800 Austria; 3 University Department of Anesthesiology, Intensive Care Medicine and Emergency Medicine, Johannes Wesling Klinikum Minden, Ruhr University Bochum, Hans-Nolte-Straße 1, Minden, 32429 Germany; 4 Coordination office for studies in biomedicine and preclinical and clinical research, Protestant Hospital of the Bethel Foundation, University Hospital OWL, University of Bielefeld, Maraweg 21, Bielefeld, 33617 Germany; 5 Department of Anaesthesiology, Intensive Care, Emergency Medicine, Transfusion Medicine and Pain Therapy, Medical School and University Medical Center OWL, Bielefeld University, Protestant Hospital of the Bethel Foundation, Burgsteig 13, Bielefeld, 33617 Germany

**Keywords:** Death, Child, Congenital, Paediatric, Prematurity

## Abstract

**Background:**

The aim of this study was to develop a risk calculation model for peri-operative 30-day-mortality in preterm infants in non-cardiac surgery.

**Methods:**

Retrospective monocentric follow-up cohort-study of 27,453 pediatric anesthesias at a German university hospital and level one perinatal center between 2008 and 2021 for non-cardiac surgeries. Inclusion criteria were age < 37 post-menstrual weeks at the time of surgery. The primary endpoint was 30-day-mortality after surgery. For statistical analysis, stepwise backwards logistic regressions were performed to identify predictors for 30-day mortality after surgery.

**Results:**

Between 2007 and 2021, 278 preterm infants underwent surgery. The 30-day-mortality was 8.6% (24/278; CI95%:5.6–12.6). A preselection of potential risk factors was based primarily on prior knowledge available from the literature and the results of previously published studies. The final prediction model using a multivariable logistic regression revealed lower post-menstrual age (odds-ratio(OR): 0.67; CI95%: 0.54–0.83) and lower body weight at the time of surgery for extremely preterm infants (OR: 0.024; CI95%: 0.003–0.22), administration of dopamine or norepinephrine or epinephrine (OR: 11.6; CI95%: 3.58–37.7), and life-threatening emergencies between 10pm-7am (OR: 10.1; CI95%: 2.36–43.5) as significant independent risk factors for 30-day-mortality. The Area-Under-The-Receiver-Operating-Characteristic-Curve (0.90; CI95%: 0.85–0.96) showed a good discrimination of the final model. The investigation of the calibration curve (*p* = 0.99, Spiegelhalter test) and the goodness of fit test (*p* = 0.85, Hosmer-Lemeshow test) indicated no significant discrepancies between estimated and observed probabilities for the peri-operative 30-day mortality.

**Conclusions:**

Peri-operative 30-day-mortality of preterm infants during non-cardiac surgery is high. The prediction model with easily ascertainable factors as described could be a valuable tool for estimating 30-day-mortality in preterm infants and should be validated in larger populations.

**Supplementary Information:**

The online version contains supplementary material available at 10.1186/s12871-025-03168-x.

## Background

In the context of anesthesiologic peri-operative care, the care of preterm infants presents a particular challenge: This unique patient population has specific anatomical and physiological characteristics. Furthermore, congenital anomalies, for example cardiac, pulmonary, and/or intestinal malformations, the immaturity of various organ systems as well as underlying diseases e.g. necrotizing enterocolitis or meconium-ileus yield a high risk for peri-operative cardiac arrest and mortality in the peri-operative setting [1–7]. 

In recent years, several risk factors for peri-operative mortality and morbidity, such as peri-operative cardiac arrest, age less than one-year, American Society of Anesthesiologists (ASA) status ≥ III, congenital defects, emergency surgery and preterm birth, have been identified in children [6–9]. Additionally, in neonatal care of preterm infants, risk factors for mortality include the degree of prematurity at birth, as well as co-factors such as infections and/or malformations [6–9]. However, these risk factors apply to almost every preterm infant, which complicates the individual risk assessment for mortality in this particular population [6–9]. Individual risk assessment is of particular importance for the attending physician, as the associated risks influence the timing and extent of surgery. From the perspective of the affected parents and their relatives, a differentiated and individualized risk assessment of the probability of a fatal outcome is important from a psychosocial point of view.

Various prognostic tools for predicting morbidity and mortality in adults show only limited validity in children. In paediatric anaesthesia the Paediatric-Risk-Assessment-Score was developed using a simplified five-variable objective score to predict mortality in neonates, infants and children undergoing non-cardiac surgery (age < 12 months, emergency surgical procedure, the presence of a neoplasm, of at least one comorbidity, and characteristics of critical illness) [10–12]. Although it was easy-to-use and an accurate tool for estimating the mortality risk, a more differentiated assessment of the mortality risk is useful with regards to preterm infants [12]. Therefore, the aim of the present follow-up-study was to develop a risk calculation tool for 30-day-mortality in the context of perioperative anesthesiologic care of preterm infants in non-cardiac surgery.

## Methods

The present study is a follow-up study of peri-operatively collected data focussing on mortality risk estimation for 30-day-mortality in preterm infants [6, 7]. The study was approved by the Institutional Review Board of the University of Muenster, Germany (file reference 2019-398-f-S). Due to its retrospective nature, the requirement of written informed consent was waived by the Institutional Review Board. The manuscript adheres to the applicable STROBE-guidelines. The anesthesia database at the Protestant Hospital of the Bethel Foundation, Medical School OWL, Bielefeld University in Germany was scanned for the timeframe from 01.01.2008–31.12.2021. All anesthesia-relevant complications from the beginning of anesthesiologic care until 60 min after completion of anesthesia and/or sedation were recorded in the database [13]. The study centre is a perinatal and national trauma center with the highest level of care in each respective speciality. It cares for about 1% of all births in the Federal Republic of Germany. The center performs all surgical procedures on children with the exception of cardiac surgery.

Only infants that were deemed preterm at the time of surgery and underwent an anesthetic procedure were included [6, 7, 13]. If multiple operations were necessary during the hospital stay, only the first operation was included. Prematurity was defined as a post-menstrual age < 37 weeks of gestation at the time of surgery. The peri-operative period was defined from the onset of anesthesiologic care until 60 min after the end of anesthesia or sedation [6, 7, 13].

Data were anonymized for evaluation. Exclusion criteria was post-menstrual age ≥ 37 weeks at time of surgery.

Demographic data (age, gestational age, post-menstrual age, age at time of surgery, sex), birth weight, weight classification according to classification of World Health Organisation (WHO) and weight on the day of anesthesia as well as existing congenital anomalies (central nervous system, airways, lungs, heart, vessels, gastrointestinal tract, kidneys) and comorbidities (central nervous system, airways, lungs, heart, vessels, blood and coagulation, gastrointestinal tract, kidneys, extremities, sepsis) according to their anatomical region were recorded. In addition, the occurrence of sepsis was recorded [6, 7].

Additionally, the pre-operative therapy with catecholamines (dobutamine, dopamine, epinephrine, norepinephrine) and the admission to the neonatal ICU were recorded.

In addition, the specific operation or intervention was recorded and assigned to a body region (intracranial, airways, thoracotomy, gastrointestinal, laparotomy, urogenital, vessels). The urgency of the surgical procedure was classified as vital (immediate), urgent (< 6 h) or elective according to surgical data. In addition, the time of day (beginning of surgery 7:01–15:00; 15:01–22:00; 22:01–07:00) and the occurrence of peri-operative cardiac arrest (POCA) and peri-operative transfusion therapy was noted. POCA was defined as any condition that required the performance of chest compressions and/or defibrillation according to Utstein-Criteria [6, 7, 14]. The indication to perform chest compressions was declared by the anaesthetist in charge. Patient outcome was evaluated and recorded 30 days after resuscitation (“dead”, “alive”).

### Statistical analysis

Data were collected in Microsoft Excel^®^ (Version 2013). SAS 9.4 (SAS Institute Inc., Cary, NC, USA) and SPSS V.29.0 (IBM, New York, New York, United States of America) were used for statistical analyses. Mortalities and incidences were shown as % or relative frequencies per 10,000 performed preterm anesthetic procedures with indication of the 95% confidence intervals (CI95%). Confidence intervals were calculated using exact Clopper-Pearson method. Results are presented as mean ± standard deviation for continuous and percentages for categorical variables. Non-parametric tests, e.g. two-tailed exact Mann-Whitney test, Fisher´s exact test and multivariable logistic regression models (backwards elimination) were performed; Receiver-Operating-Characteristic-Curves (ROC) and Area-Under-The-ROC (AUC) were calculated, and Hosmer-Lemeshow test and Spiegelhalter test were performed to check violations of goodness of fit of the logistic regression model. In addition, we performed a bootstrapping analysis (*n* = 1000) to check of the estimated regression coefficients.

## Results

Out of 27,453 a total of 392 anesthetic procedures in 278 preterm infants were performed (1.4%; 95%CI:1.3–1.6) between 01/2008 and 12/2021. There were no relevant changes of anesthetic practice or the POCA mortality rates during the observation period.

### Characteristics of preterm infants requiring surgery

All anesthetic procedures were performed in general anesthesia by specialists in anesthesia with clinical expertise in pediatric anesthesia.

According to the WHO classification, 111 (39.9%) of these children were extremely preterm (post-menstrual age < 28.weeks + 0 days), 61 (21.9%) very preterm (post-menstrual age 28. weeks + 0 days − 31. weeks + 6 days) and 106 (38.1%) were preterm (post-menstrual age 32. weeks + 0 days -≤36.weeks + 6 days). An extremely low birth weight (< 1.0 kg) was observed in 119 preterm infants (43.0%), 50 (18.1%) had a very low birth weight (1.0-<1.5 kg), 73 (26.4%) a low birth weight (1.5-<2.5 kg) and 35 (12.6%) a normal birth weight (≥ 2.5 kg).

Table 1 shows the characteristics, congenital anomalies, comorbidities, and surgical procedures of anesthesiologically treated preterm infants.

**Table 1:** Characteristics and surgical procedures of anesthesiologically treated preterm infants

	Overall [*n* = 278]	Survivor [*n* = 254]	Non-survivor [*n* = 24]	*p*-value
Male sex [n (%)]	154 (55)	143 (56)	11 (46)	0.39
Age categories (mean ± SD))				
Post-menstrual age at birth [w] (missing *n* = 2)	30.0 ± 4.5	30.2 ± 4.4	27.6 ± 4.0	**0.003**
Age at time of surgery [d]	20.5 ± 22.1	20.9 ± 22.5	16.5 ± 17.1	0.77
Post-menstrual age at time of surgery [w]	32.2 ± 3.8	32.5 ± 3.6	28.9 ± 3.6	**< 0.001**
Preterm infant [n (%)]	106 (38)	102 (40)	4 (17)	**0.02**
Very preterm infant [n (%)]	61 (22)	55 (22)	6 (25)	
Extremely preterm infant [n (%)]	111 (40)	97 (38)	14 (58)	
Body measures (mean ± SD)				
Birth weight [kg]	1.465 ± 813	1.508 ± 821	1.013 ± 564	**< 0.001**
Weight at time of surgery [g]	1.633 ± 761	1.694 ± 753	0.999 ± 532	**< 0.001**
Weight at time of surgery (kg) differentiated by preterm, very und extremely preterm				
Preterm infant	2.329 ± 505	2.343 ± 506	1.990 ± 356	0.14
Very preterm infant	1.514 ± 486	1.560 ± 487	1.112 ± 229	**0.006**
Extremely preterm infant	1.026 ± 485	1.078 ± 495	0.667 ± 154	**< 0.001**
Congenital anomalies [n (%)]	141 (51)	131 (52)	10 (42)	0.40
Central nervous system	20 (7)	20 (8)	0 (0)	0.23
Airways	26 (9)	21 (8)	5 (21)	0.06
Lungs	28 (10)	24 (9)	4 (17)	0.28
Heart	28 (10)	25 (10)	3 (12)	0.72
Vessels	11 (4)	8 (3)	3 (12)	0.06
Gastrointestinal tract	115 (41)	110 (43)	5 (21)	**0.05**
Comorbidities [n (%)]				
Central nervous system	85 (31)	72 (28)	13 (54)	**0.018**
Airways	112 (40)	99 (39)	13 (54)	0.19
Lungs	180 (65)	160 (63)	20 (83)	0.**05**
Heart	33 (12)	31 (12)	2 (8)	0.75
Blood and coagulation	112 (40)	102 (40)	10 (42)	0.99
Gastrointestinal tract	164 (59)	114 (57)	20 (83)	**0.01**
Sepsis	140 (50)	124 (49)	16 (67)	0.13
Transfer from the neonatal intensive care unit	240 (91)	218 (90)	22 (100)	0.24
Pre-Existing therapy with catecholamines [n (%)]	82 (29)	63 (25)	19 (79)	**< 0.001**
Norepinephrine	28 (10)	18 (7)	10 (42)	**< 0.001**
Dobutamine	22 (8)	15 (6)	7 (30)	**< 0.001**
Epinephrine	16 (6)	10 (4)	6 (25)	**< 0.001**
Dopamine	44 (16)	33 (13)	11 (46)	**< 0.001**
Type of surgical procedure [n (%)]				
Intracranial	40 (14)	39 (15)	1 (4)	0.22
Airways	8 (3)	8 (3)	0 (0)	0.99
Thoracotomy	33 (12)	30 (12)	3 (12)	0.99
Laparotomy	213 (77)	189 (74)	24 (100)	**0.002**
Urogenital	10 (4)	8 (3)	2 (8)	0.21
Vessels	15 (5)	12 (5)	3 (12)	0.13
Emergency categories [n (%)]				
Elective	117 (43)	114 (46)	3 (13)	**< 0.001**
Urgent	62 (23)	58 (24)	4 (17)	
Vital	90 (33)	74 (30)	16 (70)	
Time of day [n (%)]				
7:01–15:00	203 (73)	191 (75)	12 (50)	**0.004**
15:01–22:00	47 (17)	42 (17)	5 (21)	
22:01–7:00	27 (10)	20 (8)	7 (29)	
Peri-operative Cardiac Arrest [n (%)]	10 (4)	5 (2)	5 (21)	**< 0.001**
Peri-operative transfusion therapy [n (%)]				
Red blood cell concentrates	104 (38)	98 (39)	6 (25)	0.27
Fresh frozen plasma	111 (40)	105 (42)	6 (25)	0.13
Red blood cell concentrates or Fresh frozen plasma	137 (49)	128 (51)	9 (38)	0.29

*d *days, *g *gram, *SD* standard deviation, *w *weeks

p=two-sided *p*-values of the statistical tests for group comparison Survivor vs. Non-Survivor

The *p*-values should be interpreted with caution, as no adjustment of the α-error was performed. Univariate *p*-values < 0.05 should not be interpreted in terms of statistical significance but might indicate a potential predictor

The statistical evaluation was performed by two-tailed exact Mann-Whitney test or Fisher´s exact test. Emergency categories were tested by Mantel‐Haenszel Chi-square test of trend

Body weight at time of surgery was missing in *n*=3

Bold emphasize the significant *p*-values

### Peri-operative 30-day-mortality

Peri-operative 30-day-mortality in preterm infants was 8.6% (24/278; 95%CI:5.6–12.6) respectively 612.2 per 10,000 anesthesias (24/392 anesthetic procedures) in preterm infants. Peri-operative 30-day-mortality was highest in the extremely preterm infants at 12.6% (14/111; 95%CI:7.1–20.3), followed by very preterm at 9.8% (6/61; 95%CI:3.7–20.2) and preterm infants at 3.8% (4/106; 95%CI:1.0–9.4). According to the classification of birth weight, peri-operative 30-day-mortality was highest in preterm infants with extremely low birth weight at 13.4% (16/119; 95%CI:7.9–20.9), followed by preterm infants with low birth weight at 6.8% (5/73; 95%CI:2.3–15.3), very low birth weight 4.0% (2/50; 95%CI:2.3–15.3) and normal birth weight at 2.9% (1/35; 95%CI:0.1–14.9).

### Selection of potential risk factors

Potential risk factors for perinatal mortality identified in the literature (e.g. post-menstrual age at birth) and the results of univariate analysis of our data (Table 1) were used to determine a list of potential predictors of 30-day-mortality in preterm children in non-cardiac surgery.

**Fig. 1:**
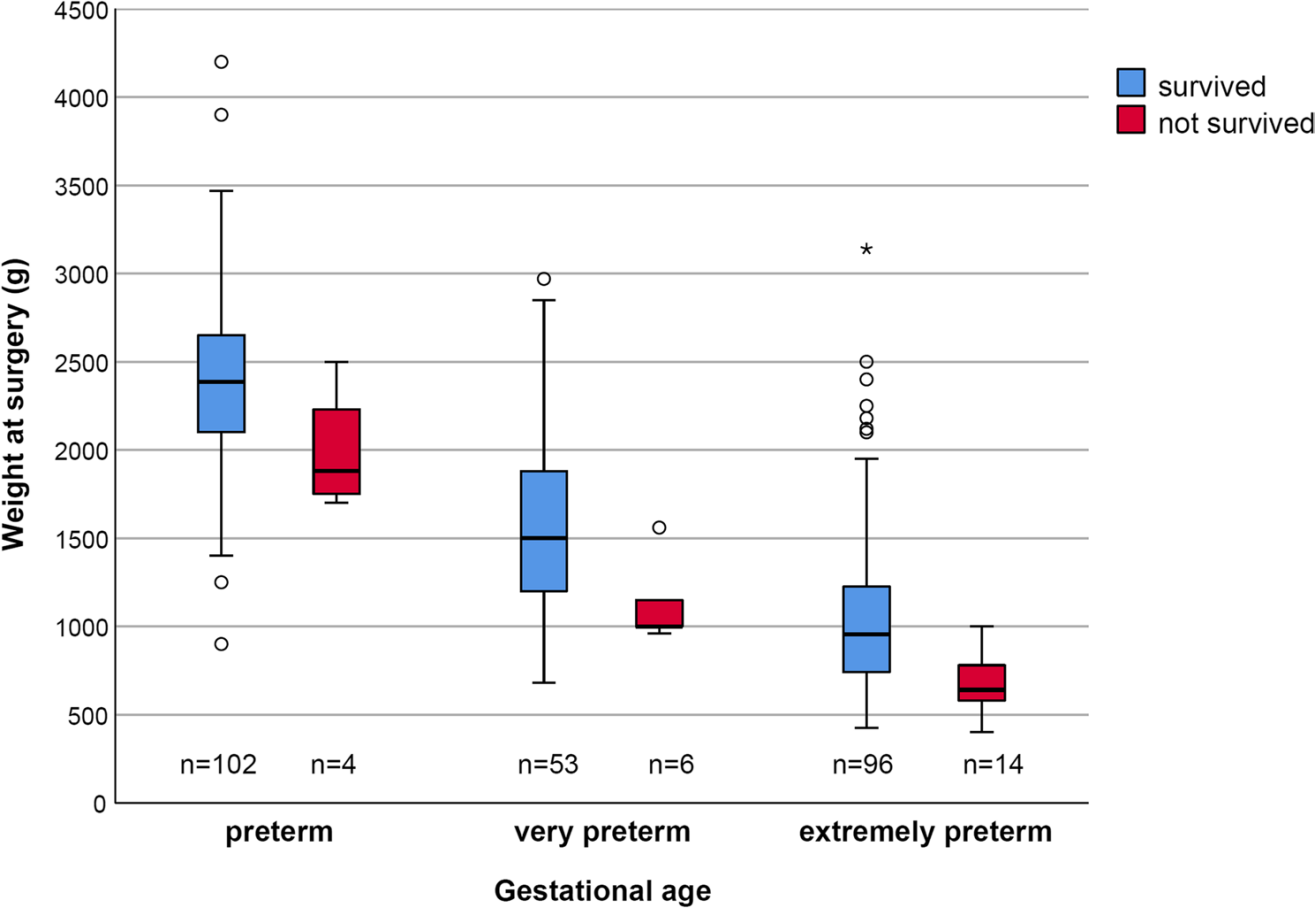
Impact of weight at the time of surgery and prematurity differentiated by perterm, very and extremely preterm on 30-day-mortality (body weight at time of surgery was missing in *n*=3)

In the previous study, lower body weight at time of surgery was identified as a strong, respectively the strongest risk factor in this patient group [6, 7]. However, Fig. 1; Table 1 indicate that the impact of body weight at the time of surgery was particularly evident in very and extremely preterm infants. Therefore, the body weight at the time of surgery was differentiated between premature, very premature and extremely premature infants. Furthermore, both the time of surgery between 10pm-7am (25.9%) and the emergency category “vital” (17.8%) were strongly associated with increased mortality. However, the vast majority (80.8%) of surgeries at night (10pm-7am) were life-threatening emergencies, these were summarized in a combined risk factor “nocturnal vital emergency” (0 = surgery between 7am-22pm *or* non-vital emergency, 1 = vital emergency between 10pm-7am). This combined risk factor has a stronger impact on mortality (28.6%; Odds Ratio [OR] = 5.27, 95%CI:1.82–15.2) than either risk factor “time of surgery” or “emergency” (see Supplement 1).

In summary, body weight at the time of birth, body weight at time or surgery (differentiated by preterm, very preterm, preterm infants), post-menstrual age at the time of birth, post-menstrual age at the time of surgery, pre-operative norepinephrine, dobutamine, epinephrine, dopamine, catecholamines, nocturnal vital emergency, and laparotomy were included as potential predictors in a stepwise (backwards, p_out_=0.15) multivariable logistic regression model. This regression model revealed post-menstrual age at time of surgery (OR:0.66; CI95%:0.53–0.83), body weight at time of surgery only in extremely preterm infants (OR:0.34; CI95%:0.004–0.326), application of dopamine (OR:4.48; CI95%:1.51–14.8), norepinephrine (OR:4.72; CI95%:1.51–14.8), the occurrence of epinephrine (OR:3.51; CI95%:0.80–15.5) and nocturnal vital emergency between 10pm-7am (OR:6.81; CI95%:1.63–28.4) as significant independent risk factors for 30-day-mortality. All included risk factors were statistically significant (*p* < 0.01), except epinephrine (*p* = 0.097). Supplement 1 shows the results in detail.

### Development of the final prediction model

The discrimination assessed with AUC under the ROC was 0.90 (CI95%:0.84–0.96). Because the number of predictors was high (*n* = 6) (in relation to the total number of infants in this study, we checked whether the prediction model could be simplified if the three predictors norepinephrine [y/n], dopamine [y/n], and epinephrine [y/n] are replaced by one combined predictor ‘(3) Catecholamines’ (norepinephrine or dopamine or epinephrine [y/n]). This simplified multivariable logistic regression model (backwards, p_out_=0.15) confirmed the significance of the combined predictor (norepinephrine or dopamine or epinephrine [y/n]) (OR: 11.6, CI95%:3.59–37.7, *p* < 0.01).

Table 2 shows the results of the final (simplified) prediction model, i.e. the multivariable logistic regression model includes four predictors: post-menstrual age at time of surgery, body weight at time of surgery only in extremely preterm infants, presurgical application of catecholamines (dopamine or norepinephrine or epinephrine), and nocturnal vital emergency.

**Table 2:** Included variables in the final multivariable logistic regression model

**Predictors**	**b**	**S.E.**	**Wald**	**Sig.**	**Odds ratio**	**95% CI odds ratio**
Post-menstrual age at time of surgery [weeks]	-.406	.110	13.746	.000	.666	.538.826
Weight at time of surgery [kg] in extremely preterm infants	-3.725	1.124	10.989	.001	.024	.003-.218
Nocturnal vital emergency	2.317	.743	9.712	.002	10.142	2.362-43.54
Preoperative Catecholamines	2.454	.601	16.694	.000	11.631	3.585-37.74
Constant	9.848	3.509	7.876	.005	n.a.	n.a.

*b* regression coefficient, *S*.*E*. standard error, *95% CI* 95% confidence interval, *n*.*a*. not applicable, Catecholamines = dopamine, norepinephrine and/or epinephrine

**Fig. 2:**
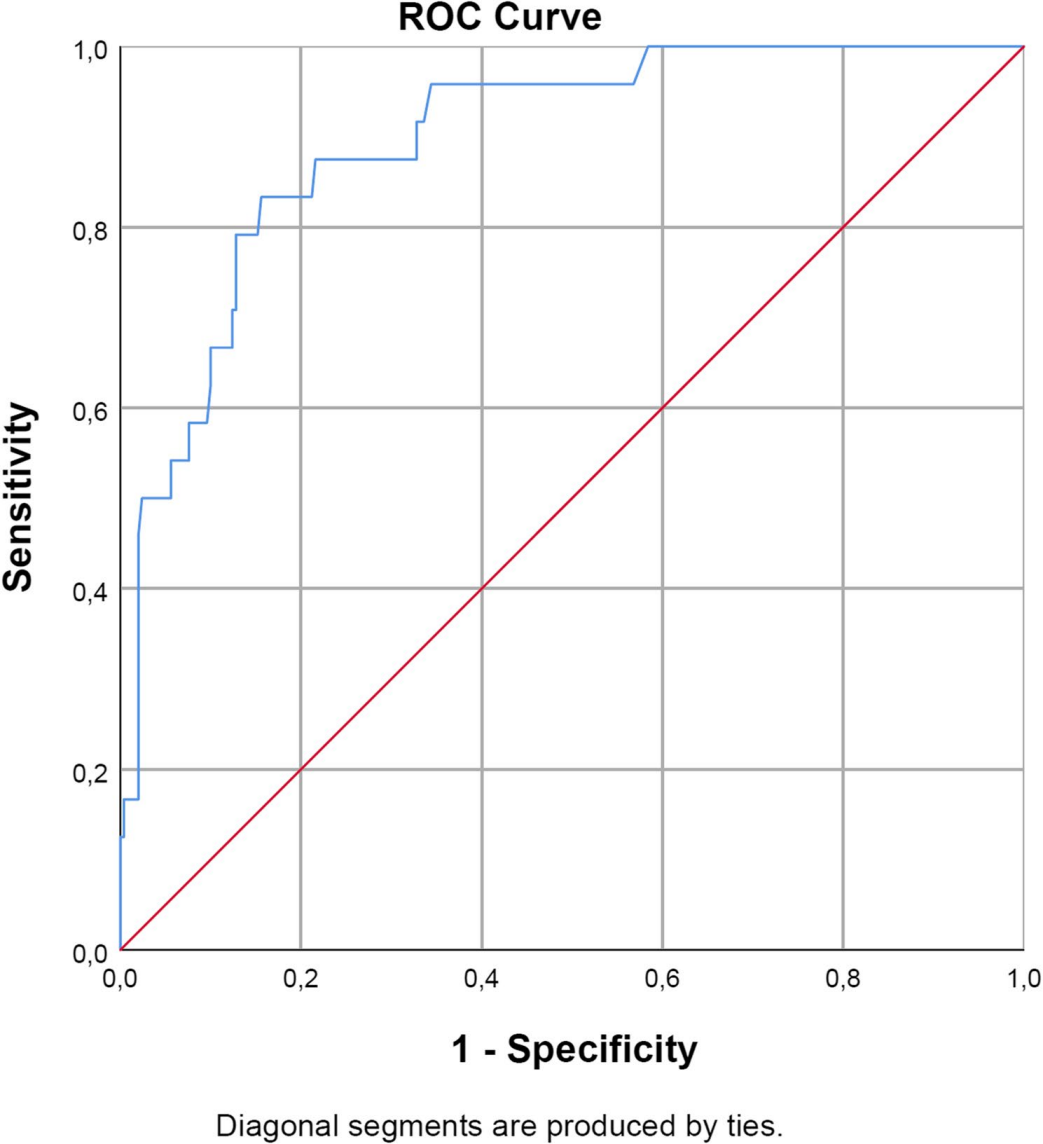
Receiver-Operating-Curve (ROC) (final model) [Area-under under the ROC = 0.90 (CI95%:0.84-0.96)]

The discrimination of this final prediction model assessed with AUC under the ROC was 0.90 (CI95%:0.84–0.96) (Fig. 2). The investigation of the calibration of the logistic regression model (visual inspection and non-significant Spiegelhalter test; *p* = 0.99) showed no significant discrepancies between estimated and observed probabilities for mortality (see Supplement 2: Calibration curve). Hosmer-Lemeshow test also indicates no significant violation of goodness of fit of the final logistic regression model (*p* = 0.84). Based on the estimated regression coefficients (Table 2), the linear predictor, abbreviated as “risk predictor” (RP), is calculated as:

$${\begin{array}{ll}\mathrm{RP}&=\;9.848\;-\;0.406{\mathrm x}_1\;- \;3.725{\mathrm x}_2\;\\&+\;2.454{\mathrm x}_3\;+\;2.317{\mathrm x}_4\end{array}}$$

X_1_ = post-menstrual age at time of surgery (weeks)

X_2_ = body weight (kg) at time of surgery in extremely preterm infants

X_3_ = pre-operative application of dopamine, norepinephrine or supra (yes = 1, no = 0)

X_4_ = nocturnal vital emergency between 10pm-7am (yes = 1, no = 0)

**Fig. 3:**
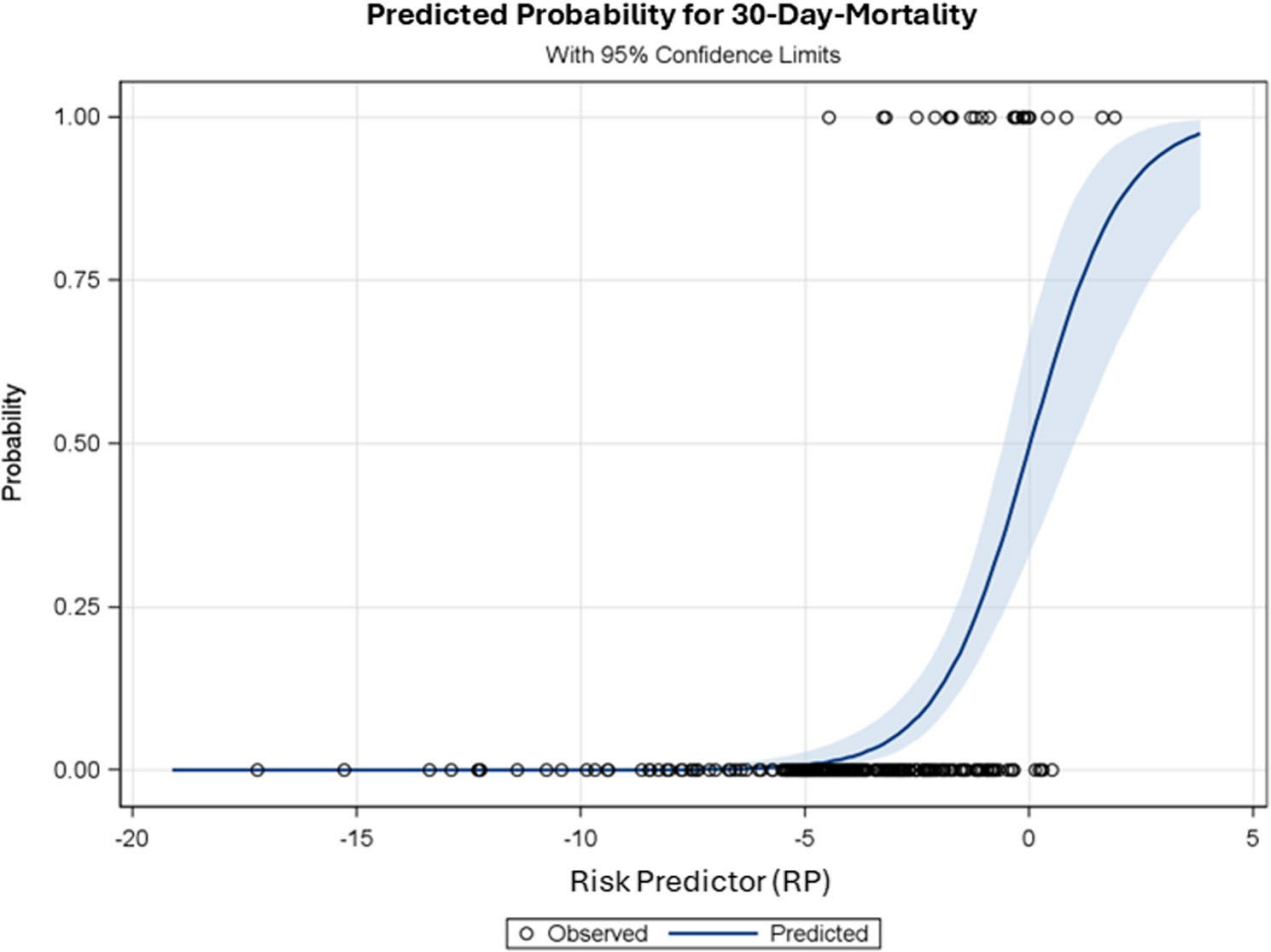
Relationship between risk predictor “RP” and the predicted probability of 30-day-mortality

According to the logistic function, the probability of 30-day-mortality is calculated as exp(RP)/(1 + exp(RP)). The relationship between the predictor RP and predicted probability of 30-day-mortality is shown in Fig. 3. In addition, the CI95%-limits indicate the accuracy of the estimated probability of 30-day-mortality.

The results of the bootstrapping procedure (significance of regression coefficients, all *p* < 0.002) and CI95% (not including ‘1’) confirmed the regression coefficients estimated by the final prediction model (Supplement 3).

**Fig. 4:**
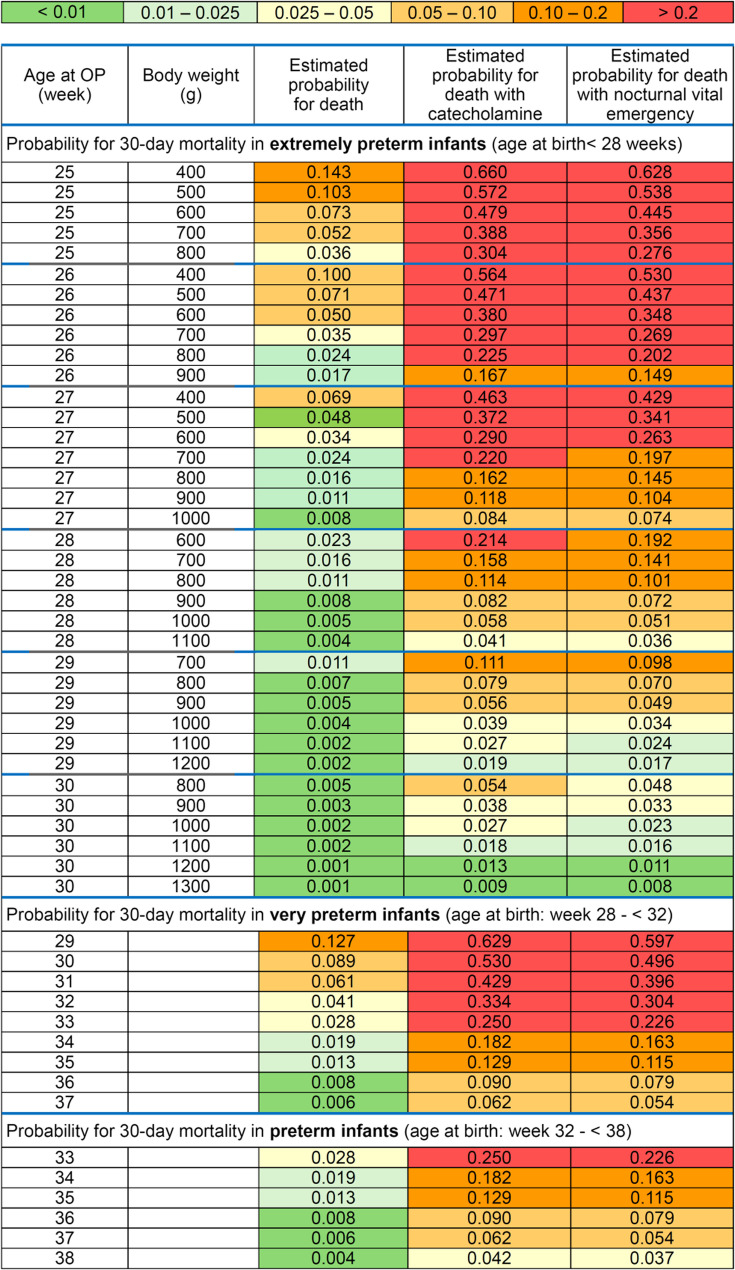
Probability for 30-day mortality

The probabilities for peri-operative 30-day-mortality differentiated by extremely preterm, very preterm and preterm infants are shown in Fig. 4. Figure 4 illustrates the influence of the individual factors, e.g. catecholamines, on mortality. In particular, Fig. 4 can be used to roughly estimate the probability of mortality without tools (such as calculators, etc.).

### 30-day-mortality in extremely preterm infants (age at birth < 28 weeks)

The 30-day mortality rate was highest in this group at 12.6% (14/111). 12 (85.7%) of the 14 deceased infants were treated with dopamine, norepinephrine or epinephrine before surgery and 4 (28.6%) as a nocturnal vital emergency. Of the 97 survivors, 35 (36.1%) were treated with dopamine, norepinephrine or epinephrine and 10 (10.3%) as nocturnal vital emergencies. However, it is worth noting that from a gestational age > 30 weeks at the time of surgery, the probability of death in this group was very low.

### 30-day-mortality in very preterm infants (age at birth: week 28 - < 32)

The 30-day mortality rate in this group was 9.8% (6/61). 5 (83.3%) of the 6 deceased infants were treated with dopamine, norepinephrine or epinephrine before surgery and none was treated as nocturnal vital emergency. Of the 55 survivors, (only) 4 (7.3%) were treated with dopamine, norepinephrine or epinephrine and 3 (5.7%) as nocturnal vital emergencies.

### 30-day-mortality in preterm infants (age at birth: week 32 - < 38)

The 30-day mortality rate was lowest in this group 3.8% (4/106). 1 (25%) of the 4 deceased infants was treated with dopamine, norepinephrine or epinephrine before surgery and 2 (50%) were treated as nocturnal vital emergencies. Of the 102 survivors, only 7 (6.9%) were treated with dopamine, norepinephrine or epinephrine and only 2 (2.0%) were treated as nocturnal vital emergencies.

## Discussion

The present study investigated 30-day-mortality and its risk factors in 278 preterm infants in a German level-one-perinatal-center. 30-day-mortality was 8.6%. The multivariable prediction model showed that lower post-menstrual age and lower body weight at time of surgery especially in extremely preterm infants, application of catecholamines like dopamine, application of norepinephrine, and nocturnal vital emergency between 10pm-7am were important risk factors for predicting mortality. The prediction model showed a good discrimination between survival and death within 30 days after surgery. The calibration curve showed no significant discrepancies between estimated and observed probabilities for mortality.

Despite the progress in peri-natal medicine in recent years, the morbidity and mortality of preterm children has remained high: Within the first year of life for children peri-operative 30-day-mortality ranges between 0 and 180 per 10,000 pediatric anesthesias. Particularly high mortality rates could be observed when considering only preterm infants. Common serious congenital anomalies and/or perinatal complications requiring surgical care make this a high risk patient collective [6, 7, 13, 15, 16]. In the prospective multicenter NECTARINE-study, which investigated morbidity and mortality after anesthesia in neonates and children in 165 centers in 31 european countries, a 30-day-mortality of 4.1% was found in the subpopulation of neonates < 28 days post birth, mainly caused by sepsis and multiorgan failure [17]. Although NECTARINE showed that the relative risk of 30-day-mortality increased with decreasing post-menstrual age, NECTARINE does not provide any differentiated information on the mortality in the group of preterm children within the different categories [17]. Therefore, the present data gives an insight into the outcomes of neonatal surgery at a German university hospital with approximately 20 preterm neonates per year or one every other week in the real world, confirming the existing risk factors in this unique population and therefore is an interesting addition to previous studies such as NECTARINE.

Various risk factors influencing peri-operative mortality in children have been described which apply to preterm infants in the peri-operative setting anyway: ASA physical status, neonatal age, age at surgery, congenital anomalies, comorbidities e.g. sepsis, preoperative organ dysfunction and organ support, preoperative blood transfusion, emergency category of the surgery, surgery in the off hours, pre-operative and peri-operative cardiac arrest as well as the presence of a do-not-resuscitate order and many others. However, some of these risk factors only manifest themselves in the intra- or post-operative course and are therefore not available pre-operativelyIn addition, it is sometimes difficult for relatives to interpret effect estimates, especially if several risk factors are present in combination [18–26]. 

Therefore, a differentiated multivariable prediction model for peri-operative mortality appears to be useful for various reasons: From the parents/guardians´ perspective an appropriate prognosis assessment tool enables a transparent understanding of risk with realistic expectations of the post-operative outcome, the definition of treatment limits, and, if necessary, alleviate feelings of anxiety as well as psychological support and the preparation of religious rituals. With regards to the healthcare professionals involved, an improved assessment of 30-day-mortality and associated risk factors may facilitate care of this very special cohort. It may provide increased precision of the prognostic evaluation and streamline the assessment of procedural risk. Furthermore, precise risk estimation ensures the allocation of adequate resources such as monitoring, equipment and the presence of experienced specialists along with appropriate post-operative follow up care [27–29]. 

For neonatal intensive care several prognostic calculators have been developed to estimate mortality. However, although the AUC in these scores ranged from to 0.96 their transferability is limited because they were not evaluated in the peri-operative setting [18–26]. Therefore, the present prediction model evaluates peri-operative mortality risk in a more differentiated manner, focusing on the peri-operative care of preterm infants in the context of non-cardiac surgery based on factors that are easy to collect combining the peri-operative risk with the risk of neonatal intensive care, demonstrating a good discrimination (AUROC:0.90; CI95%:0.84–0.96). Since an excessive variety of variables to be collected is likely to increase the accuracy of the assessment tool, but also reduces user-friendliness, one advantage of the risk assessment tool on which the present work is based, is the assessment on the basis of simple factors to be collected in a manageable framework at the bedside [30]. User-friendliness can be increased by programming an application, like the American College of Surgeons NSQIP Pediatric Surgical Risk Calculator, or a special calculator programmed in the electronic patient file [31]. 

### Limitations

The limitations of this study include its retrospective and monocentric design. Since the university hospital conducting the study is a perinatal center with the highest level of care and one of the biggest pediatric surgery centers in Germany, it is quite possible that local expertise and higher standards could have distorted the value. Although preterm infants in Germany are being treated at such centres, the present study may not be representative of the general population of preterm infants undergoing surgery. So far, no external validation of the developed prediction model has been carried out. Ideally this should be carried out as part of a multicentric international study. However, the perioperative care of preterm infants is rare, as was already demonstrated in NECTARINE. Although only a few risk scores for children have been externally validated so far, the publication of this prediction model may encourage some centres to provide validation. It may also be possible to use this validation to develop a scoring system in order to weigh the various influencing factors against each other. The present prediction model was not developed for the prediction of mortality in children undergoing cardiac surgery, traumatized children, or children undergoing solid organ transplant. These specific patient populations need a specific risk assessment tool and special validation. The present study only evaluates the prediction of mortality, so that morbidity is not taken into account. Possibly, the use a combination of mortality and major morbidity as the outcome, could increase the applicability of the predictive model and the clinical value. Furthermore, the small number of preterm infants may have biased the results. However, the number of preterm infants even in large and international multicenter studies is typically low. Accordingly, the fact that the present study is the largest within the collective of preterm infants to date must be recognized.

## Conclusions

Lower post-menstrual age and body weight at time of surgery, especially in extremely preterm infants, application of catecholamines like dopamine, norepinephrine or norepinephrine and nocturnal vital emergency are relevant risk factors for 30-day-mortality. The prediction model “PrEdiction of MortAlity in peri-operative Care of preterm cHildren” (PEACH) developed on the basis of these influencing factors could prove to be important for peri-operative risk assessment, rational risk education for parents, optimizing timing of surgery and ensuring optimum peri-operative care by the experienced specialist teams.

## Supplementary Information

Supplementary Material 1. Variables included the risk model after backward elimination.

Supplementary Material 2. Calibration for 30-day-mortality.

Supplementary Material 3. Results of the bootstrapping analysis.

## Data Availability

The datasets used and/or analysed during the current study are available from the corresponding author on reasonable request.

## Abbreviations

AUC: Area-Under-The-ROC
CI95%: 95% confidence intervals
ICU: Intensive Carew Unit
OR: Odds Ratio
POCA: Peri-operative Cardiac Arresr
ROC: Receiver-Operating-Characteristic-Curves
WHO: World Health Organisation
