# Supplementary material for: Prediction of peri-operative mortality in care of preterm children in non-cardiac surgery
Source: BMC Anesthesiol. 2025 Jun 19;25:296. doi: 10.1186/s12871-025-03168-x (PMC12180206; doi:10.1186/s12871-025-03168-x)
Supplement: Supplementary file 1 — Supplementary Material 1. Variables included the risk model after backward elimination. [file 12871_2025_3168_MOESM1_ESM.docx]

**Supplement 1:** Variables included the risk model after backward elimination

|  | **B** | **S.E.** | **Wald** | **df** | **Sig.** | **Odds Ratio** | **CI95% for Odds Ratio** |
| --- | --- | --- | --- | --- | --- | --- | --- |
| **Post-menstrual age at time of surgery [w]** | -0.413 | 0.114 | 13.244 | 1 | 0.000 | 0.662 | 0.530-0.826 |
| **Weight [kg] at time of surgery in in extremely preterm infants** | -3.375 | 1.150 | 8.620 | 1 | 0.003 | 0.034 | 0.004-0.326 |
| **Norepinephrine** | 1.552 | 0.583 | 7.085 | 1 | 0.008 | 4.723 | 1.506-14.82 |
| **Epinephrine** | 1.256 | 0.757 | 2.753 | 1 | 0.097 | 3.512 | 0.796-15.49 |
| **Dopamine** | 1.500 | 0.576 | 6.779 | 1 | 0.009 | 4.480 | 1.449-13.85 |
| **Emergency surgery** | 1.918 | 0.729 | 6.914 | 1 | 0.009 | 6.806 | 1.630-28.43 |
| **Constant** | 10.29 | 3.618 | 8.097 | 1 | 0.004 | Not applicable | Not applicable |
| Variable(s) entered on final step 7: Post-menstrual age at time of surgery [w], Weight [kg] at time of surgery in extremely preterm infants, Norepinephrine, Epinephrine, Dopamine, Emergency surgery | | | | | | | |
