# Supplementary figures and images for: Prediction of peri-operative mortality in care of preterm children in non-cardiac surgery
Source: BMC Anesthesiol. 2025 Jun 19;25:296. doi: 10.1186/s12871-025-03168-x (PMC12180206; doi:10.1186/s12871-025-03168-x)

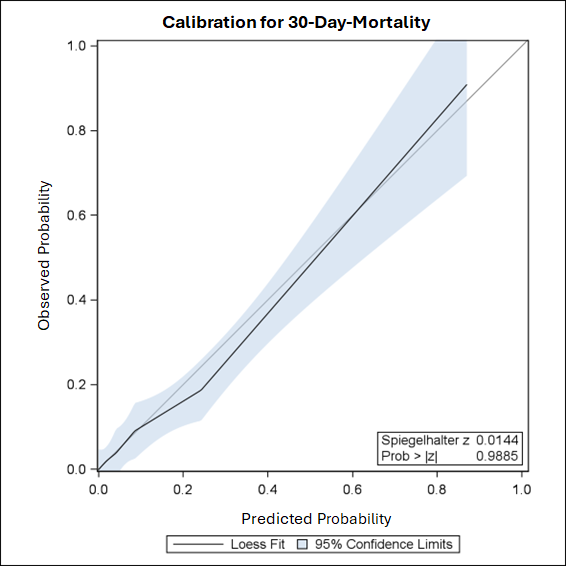

Supplement: Supplementary file 2 — Supplementary Material 2. Calibration for 30-day-mortality. [file 12871_2025_3168_MOESM2_ESM.tif]
