# Supplementary material for: Prediction of peri-operative mortality in care of preterm children in non-cardiac surgery
Source: BMC Anesthesiol. 2025 Jun 19;25:296. doi: 10.1186/s12871-025-03168-x (PMC12180206; doi:10.1186/s12871-025-03168-x)
Supplement: Supplementary file 3 — Supplementary Material 3. Results of the bootstrapping analysis. [file 12871_2025_3168_MOESM3_ESM.docx]

**Supplement 3:** Results of the bootstrapping analysis

|  | **B** | **Distortion** | **S.E.** | **p value**  **(two-sided)** | **CI95%**  **B** | **BCa 95%CI for Odds Ratio** |
| --- | --- | --- | --- | --- | --- | --- |
| **Post-menstrual age at time of surgery [weeks]** | -0.406 | -0.042 | 0.128 | 0.001 | -0.648, -0.290 | 0.523, 0.748 |
| **Weight at time of surgery [kg] in extremely preterm infants** | -3.725 | -0.390 | 1.330 | 0.001 | -06.17, -2.25 | 0.002, 0.106 |
| **Preoperative Catecholamines** | 2.454 | 0.203 | 0.811 | 0.001 | 1.13, 4.69 | 3.09, 108.7 |
| **Nocturnal vital emergency** | 2.317 | 0.111 | 1.097 | 0.002 | 0.309, 4.51 | 1.36, 90.7 |
| **Constant** | 9.848 | 1.132 | 4.020 | 0.002 | 2.68, 23.4 | n.a. |
| Legend | BCa = bias-corrected and accelerated methode  B = regression coefficient; S.E.= standard error;  95% CI= 95% confidence interval; n.a.= not applicable;  Catecholamines = dopamine, norepinephrine, or epinephrine  The bootstrapping analysis (n=1,000) was performed by the bias-corrected and accelerated- (BCa) methode to check the estimated regression coefficients of the final regression model. The results of the bootstrapping (significance of regression coefficients, all p<0.002) and BCa 95% of OR (not including ‘1’)) are in agreement with the estimated regression coefficients. | | | | | |
